# Differential association between inflammatory cytokines and multiorgan dysfunction in COVID-19 patients with obesity

**DOI:** 10.1371/journal.pone.0252026

**Published:** 2021-05-26

**Authors:** Marie-Agnès Dragon-Durey, Xiaoyi Chen, Amos Kirilovsky, Nadine Ben Hamouda, Carine El Sissy, Jules Russick, Etienne Charpentier, Yannick Binois, Florence Marliot, Maxime Meylan, Clémence Granier, Hélène Pere, Antonin Saldmann, Bastien Rance, Anne Sophie Jannot, Stéphanie Baron, Mouna Chebbi, Antoine Fayol, Nathalie Josseaume, Claire Rives-Lange, Pierre-Louis Tharaux, Bernard Cholley, Jean-Luc Diehl, Jean-Benoît Arlet, Michel Azizi, Alexandre Karras, Sébastien Czernichow, David M. Smadja, Jean-Sébastien Hulot, Isabelle Cremer, Eric Tartour, Elie Mousseaux, Franck Pagès

**Affiliations:** 1 Laboratory of Immunology; 2 Hôpital Européen Georges Pompidou, AP-HP, Paris, France; 3 Université de Paris, Paris, France; 4 INSERM UMRS 1138, Cordeliers Research Center, Team Inflammation, Complement, and Cancer, Paris, France; 5 Sorbonne Université, Cordeliers Research Center, Paris, France; 6 INSERM UMRS 1138, Cordeliers Research Center, Team Information Sciences to Support Personalized Medicine, Paris, France; 7 Laboratory of Information Sciences to support Personalized Medicine, Paris, France; 8 INSERM UMRS 1138, Cordeliers Research Center, Team Integrative Cancer Immunology, Paris, France; 9 Department of Radiology; 10 Department of Nephrology; 11 INSERM, Paris Cardiovascular Center / PARCC, UMR 970, Paris, France; 12 Laboratory of Virology; 13 Biostatistics and Public Health Department; 14 Department of Physiology; 15 Clinic Investigation Center 1418; 16 Department of Nutrition; 17 Department of Intensive Medicine, Reanimation; 18 INSERM UMR-S1140, Team Innovative Therapies in Haemostasis, Paris, France; 19 Department of Internal Medicine; 20 Department of Vascular Medicine; 21 Department of Hematology; Heidelberg University Hospital, GERMANY

## Abstract

To investigate the mechanisms underlying the SARS-CoV-2 infection severity observed in patients with obesity, we performed a prospective study of 51 patients evaluating the impact of multiple immune parameters during 2 weeks after admission, on vital organs’ functions according to body mass index (BMI) categories. High-dimensional flow cytometric characterization of immune cell subsets was performed at admission, 30 systemic cytokines/chemokines levels were sequentially measured, thirteen endothelial markers were determined at admission and at the zenith of the cytokines. Computed tomography scans on admission were quantified for lung damage and hepatic steatosis (n = 23). Abnormal BMI (> 25) observed in 72.6% of patients, was associated with a higher rate of intensive care unit hospitalization (p = 0.044). SARS-CoV-2 RNAaemia, peripheral immune cell subsets and cytokines/chemokines were similar among BMI groups. A significant association between inflammatory cytokines and liver, renal, and endothelial dysfunctions was observed only in patients with obesity (BMI > 30). In contrast, early signs of lung damage (ground-glass opacity) correlated with Th1/M1/inflammatory cytokines only in normal weight patients. Later lesions of pulmonary consolidation correlated with BMI but were independent of cytokine levels. Our study reveals distinct physiopathological mechanisms associated with SARS-CoV-2 infection in patients with obesity that may have important clinical implications.

## Introduction

Coronavirus disease 2019 (COVID-19), first identified on December 2019, is caused by the severe acute respiratory syndrome coronavirus-2 (SARS-CoV-2) [1, 2]. Acute respiratory distress syndrome (ARDS) sometimes associated with multiorgan failure is observed in a large proportion of COVID-19 patients [3]. The pathogenesis of severe forms of COVID-19 is characterized by hyperinflammatory syndrome, i.e. a cytokine storm [4]; with overproduction of inflammation-related molecules such as interleukin-6 (IL-6), C-reactive protein (CRP), and ferritin [5].

If low socioeconomic status [6] has been reported as risk factors for being infected, severe form of COVID-19 is mainly observed in patients of advanced age; with the 14.8%-20.2% fatality rate for adults older than 80 years [7]. However, it also affects younger male patients with preexisting moderate comorbidities i.e. type II diabetes (33.8%), high blood pressure (56.6%), and overweight (41.7%), in US patients requiring hospitalization [8]. The association between high body mass index (BMI) and the need for invasive mechanical ventilation has been emphasized by studies from different countries [9–12]. This view is endorsed by a position statement concerning the prevention and management of patients with obesity infected by SARS-CoV-2 [13]. Obesity is considered as a state of excessive fat accumulation caused by a disruption of energy balance. It is marked by enhanced pro-inflammatory factors in blood and infiltration of immune cells in white adipose tissue. Imbalance in the expression of pro and anti-inflammatory adipokines secreted by adipose tissue contributes to the development of obesity-linked complications [14], e.g. insulin resistance and type II diabetes, atherosclerosis, and non-alcohol fatty liver disease (NAFLD) [15]). The interleukin (IL)-1-family cytokine members and tumor necrosis factor alpha (TNFα) are key inflammatory cytokines involved in fatty liver diseases [16, 17]. In addition, other cytokines, such as MCP-1, IL-6, IL-8, IL-18, RANTES and Il-10 are also associated to fatty liver [18, 19]. Pro-inflammatory responses in adipose microenvironment activate endothelial cells, which upregulate cell adhesion markers such as P and E-selectins [20] favoring infiltration of immune cells. Signs of liver dysfunction [21], acute kidney injury [22], and endothelium activation [23] have been reported in critically ill patients with COVID-19, however their association with obesity and inflammatory disorders remains elusive.

In the present study, we analyzed prospectively the association between the COVID-19-induced cytokines storm and vital organs, i.e. the lung, liver, and kidney in 51 severe patients according to their BMI and examined whether the severity of the SARS-CoV-2 infection in overweight patients could be related to distinct cytokine profiles and/or a differential impact of inflammatory cytokines on multiorgan dysfunction.

## Materials and methods

### Patients

This prospective observational cohort study included 51 consecutive adult patients (≥ 18 years old) with available samples admitted to Georges Pompidou European Hospital (Paris, France) since March 23^rd^ 2020. All patients were diagnosed with COVID-19, i.e. positive for SARS-CoV-2 nucleic acid on real-time reverse transcription-polymerase chain reaction (RT-PCR) assays of nasopharyngeal swab specimens, in accordance with the World Health Organization (WHO) COVID-19 technical guidance (https://apps.who.int/iris/handle/10665/330854). Only critical cases of pneumonia defined as patients with respiratory rate > 30 breaths/min, severe respiratory distress, or SpO2 < 90% on room air at admission were included in this study. On admission, all patients required oxygen and 36 an intensive care unit (ICU) admission with invasive mechanical ventilation. Five patients received corticosteroids at admission and 17 anti-IL6R during hospitalization. This study received approval from the hospital institutional review board (CERAPHP.5—Comité d’Ethique de la Recherche, Assistance Publique Hôpitaux de Paris, registration number of the committee: N° IRB #00011928, N° approval: 2020-CVD-03). All patients provided written informed consent for study participation prior to inclusion. Patients included in the present study were adult and informed that their medical data could be used for research purpose in accordance with the General Data Protection Regulation (EU 2016/679).

### Data collection

Epidemiological, demographic, clinical, laboratory, treatment, and outcome data were extracted from patients’ electronic medical records and collected in the dedicated HEGP- REDCap^™^ database, which has been registered under the MR004 CNIL procedure. All data were reviewed by physicians (JSH, JLD), radiologists (EM, EC), virologist (HP), hematologist (DMS), immunologists (MADD, ET, FP), and biostatisticians (ASJ, BR, XC, MM, AK).

### Routine laboratory tests

Routine laboratory tests on admission and during follow-up included the levels of liver enzymes: alanine aminotransferase (ALT), aspartate aminotransferase (AST), gamma-glutamyltransferase (GGT), alkaline phosphatase, and total bilirubin (TBIL). Hepatic steatosis index (HSI) was calculated by adding an ALT/AST ratio to BMI, with two points added to the algorithm for diabetes and two for females [22]. Kidney parameters included baseline creatinine values, the peak value of serum creatinine during follow-up (creatinine Max), the urine albumin/creatine ratio (ACR), the urine protein/creatine ratio (PCR), and the urinary concentration of sodium (UNa) and potassium (UK) from spot urine samples collected during the initial 48-h following admission.

#### Computed tomography (CT) examination and analysis

Twenty-three patients had available chest CT scan at admission (15 patients in conventional hospitalization unit and eight in ICU). Visual assessment of lung injuries was performed independently of care by two expert radiologists blinded to all clinical and biology data (EM, ET). Lung lobes were assessed for the presence of either ground-glass opacity (hazy areas of increased attenuation without obscuration of the underlying vasculature; GGO) or consolidation (homogeneous opacification with obscuration of the underlying vasculature), or both in all patients [24, 25]. The extent was further evaluated by the number of affected lobes (0 to 5). In case of discordance, a consensus was reached by the two experts before calculating the final visual GGO and consolidation scores. As previously described [26], hepatic steatosis was screened in all CTs by estimating the absolute value difference in attenuation between liver and spleen (CTL-S). A Threshold CTL-S value of -3.2 was used to define the presence of hepatic steatosis. A coronal height measurement of the hepatic hilum at the dome level was finally performed in all patients at CT.

#### Serum SARS-CoV-2 nucleic acid (RNAaemia)

SARS-CoV-2 RNA was extracted from plasma (140μL) collected at the zenith of cytokines concentrations, using QIAamp^®^ Viral RNA Mini Kit (QIAGEN^®^, Hilden, Germany), according to the manufacturer’s instructions. SARS-CoV-2 RNAaemia was quantified by droplet-based Crystal Digital PCR^™^ (Stilla Technologies, Villejuif, France) on the Naica^™^ System (Stilla Technologies, Villejuif, France) using the commercial RT-PCR amplification kit (Novel Coronavirus (2019-nCoV) Digital PCR Detection Kit, Apexbio^™^, Beijing, China) following the manufacturer’s instructions. Plasma samples with one of the two ORF1 or N genes or both genes detected were considered as positive samples and results were automatically analyzed using "Crystal reader" (Stilla) and "Crystal Miner" software (Stilla) based on the most amplified gene positive droplets. SARS-CoV-2 RNA concentrations (cp/mL) were finally calculated considering the extracted volume of plasma.

#### Flow cytometry

Peripheral blood mononuclear cells collected at admission were stained for CD3, DC-SIGN (Beckman Coulter, Brea, CA, USA) CD8, CD40, HLA-DR (BioLegend, San Diego, CA, USA), CD14, CD16, CD19, CD56, CD86, CD209 (BD Biosciences), CD163 (Miltenyi, Bergisch Gladbach, Germany), and Live/Dead Fixable Yellow (Thermofisher, Waltham, MA, USA) (details in S1 Table). Data were acquired on a Fortessa X20 flow cytometer (BD Biosciences) and analyzed were using Diva software (BD Biosciences). The Excyted pipeline was used to normalize and cluster the data (https://github.com/maximemeylan/Excyted). Intensity values of events gated from live cells were normalized using the Logicle transformation. Unsupervised clustering and Uniform Manifold Approximation and Projection (UMAP) were computed with 10.000 events for each sample using k = 30 [27].

#### Cytokines and chemokines measurements

The levels of 26 cytokines/chemokines (FGF2, Eotaxin, G-CSF, GM-CSF, MCP-1, MIP-1α, MIP-1β, PDGF-BB, RANTES, VEGF, IFN-γ, IL-1β, TGFβ, IL-2, IL-4, IL-5, IL-6, IL-7, IL-8, IL-9, IL-10, IL-12p70, IL-13, IL-15, IL-17A, IP-10) and two immune-related molecules (IL-1ra, sCD25) were measured by Luminex technology (Bio-Plex, Bio-Rad, 27-Plex Assays panel, Marnes-la-Coquette, France) according to the manufacturer’s instructions, in the patients plasma collected at days 1 (admission), 3, 6, 9, and 14. In patients treated with anti-IL6R therapy (17/51), only samples before immunotherapy were analyzed. Plasma samples from 18 adult blood donors collected during the same period were used as controls. Plasma HO-1 and Neopterin concentrations were determined at day 1 by using enzyme-linked immunosorbent assay (ELISA) kits (Abcam, The Netherlands and IBL International, Germany, respectively) according to the user manuals.

#### Endothelial markers measurements

Soluble VCAM-1, E-selectin, P-selectin, VEGF-A, PlGF, basic-FGF, VEGFR-2, angiopoietin-1, angiopoietin-2, and endoglin were quantified at admission on the day of the cytokine peak in PPP with a Human Magnetic Luminex Assay from R&D systems (Lille, France). Data were assessed with the Bio-Plex 200 using the Bio-Plex Manager 5.0 software (Bio-Rad, Marnes-la-Coquette, France). Circulating endothelial cells were isolated from EDTA blood samples collected at day 14 by immunomagnetic separation with mAb CD146-coated beads and stained with the fluorescent probe acridine orange, as previously described [28].

### Statistics

The association between BMI categories and patient clinical characteristics was assessed by the chi-squared test and Fisher’s exact test for categorical parameters or the Kruskal-Wallis test for continuous parameters. The p-value for trend was computed from the Spearman’s test for continuous parameters or the Mantel-Haenszel linear-by-liner association test for categorical parameters. For all pairwise comparisons the Wilcoxon-Mann-Whitney test was used followed by the Benjamini Hochberg test for multiple testing correction. In each BMI group, the median ratios between patients and the concentrations of cytokines in healthy donors were displayed on each axes of a radar chart using the radarchart function from the fmsb package. The correlations between patient clinical parameters and cytokine concentrations were assess through the Pearson correlation test except for lung damages analysis where the Spearman’s rank correlation coefficient was computed and visualized in heatmaps. For all boxplots, the center was drawn through the median of the measurement, and the lower and upper bounds of the box corresponded to the first and third quartile. Whiskers beyond these points represented 1.5 times the interquartile range. All analyses were performed with the statistical software R version 3.6.3 using gplots, ggplot2, ggpubr, and fmsb packages. A p value less than 0.05 was considered as significant.

## Results

### Clinical and biological characteristics

We prospectively collected data regarding clinical symptoms and outcomes for 51 hospitalized patients with confirmed COVID-19. Baseline clinical characteristics are shown in Table 1.

**Table 1:** Demographic and clinical characteristics.

	All	Body Mass Index			p overall	p trend
		< 25	25 < BMI < 30	≥ 30		
	N = 51	n = 14	n = 24	n = 13		
**Sex (%)**					0.637	0.579
Female	12 (23.5)	2 (14.3)	7 (29.2)	3 (23.1)		
Male	39 (76.5)	12 (85.7)	17 (70.8)	10 (76.9)		
**Age, years**	60.0 (53.0–70.5)	64.5 (59.2–75.2)	59.0 (53.8–68.0)	58.0 (49.0–68.0)	0.174	0.069
**Time from clinical signs to admission, days**	7.0 (6.0–8.5)	7.00 (6.2–7.7)	7.00 (6.0–7.2)	7.0 (7.0–10.0)	0.571	0.524
**ICU required on admission (%)**					0.526	0.258
No	15 (29.4)	6 (42.9)	6 (25.0)	3 (23.1)		
Yes	36 (70.6)	8 (57.1)	18 (75.0)	10 (76.9)		
**ICU required during hospitalization (%)**					0.084	**0.044**
No	8 (15.7)	5 (35.7)	2 (28.3)	1 (7.7)		
Yes	43 (84.3)	9 (64.3)	22 (97.1)	12 (92.3)		
**Death (%)**					0.109	0.897
No	35 (68.6)	8 (57.1)	20 (83.3)	7 (53.8)		
Yes	16 (31.4)	6 (42.9)	4 (16.7)	6 (46.2)		
**Comorbidities**						
**Diabetes (%)**					0.053	**0.024**
No	37 (72.5)	12 (85.7)	19 (79.2)	6 (50.0)		
Yes	14 (27.5)	2 (14.3)	5 (20.8)	6 (50.0)		
**AHT (%)**					0.782	0.833
No	23 (45.1)	6 (42.9)	12 (50.0)	6 (46.2)		
Yes	28 (54.9)	8 (57.1)	12 (50.0)	7 (53.8)		
**Cardiac disease other than AHT (%)**					0.12	**0.032**
No	44 (86.3)	10 (71.4)	21 (87.5)	13 (100.0)		
Yes	7 (13.7)	4 (28.6)	3 (12.5)	0 (0.0)		
**AHT, diabetes, or other cadiac disease (%)**					0.197	0.291
No	18 (35.3)	5 (35.7)	11 (45.8)	2 (15.4)		
Yes	33 (64.7)	9 (64.3)	13 (54.2)	11 (84.6)		
**COPD (%)**					0.069	**0.029**
No	47 (92.2)	14 (100.0)	23 (95.8)	10 (76.9)		
Yes	4 (7.8)	0 (0.0)	1 (4.17)	3 (23.1)		
**Cancer (%)**					0.432	0.116
No	48 (94.1)	12 (85.7)	23 (95.8)	13 (100.0)		
Yes	3 (5.9)	2 (14.3)	1 (4.2)	0 (0.0)		
**Tabacco use (%)**					1	1
No	27 (90.0)	7 (87.5)	13 (92.9)	7 (87.5)		
Yes	3 (10.0)	1 (12.5)	1 (7.1)	1 (12.5)		
**Therapy**						
**Corticotherapy**^A^ **(%)**					1.000	0.950
No	46 (90.2)	13 (92.9)	21 (87.5)	12 (92.3)		
Yes	5 (11.9)	1 (7.7)	3 (15.8)	1 (10.0)		
**Anti-IL-6R (Sarilumab)**^B^ **(%)**					0.597	0.345
No	34 (66.7)	11 (78.6)	15 (62.5)	8 (61.5)		
Yes	17 (33.3)	3 (21.4)	9 (37.5)	5 (38.5)		

ICU = Intensive care unit, COPD = Chronic obstructive pulmonary disease, AHT = arterial hypertension

^A^ Patients received corticotherapy at admission and during hospitalization.

^B^ Patients received anti-IL6R treatment during hospitalization, none received it at admission.

In total, 36 and 7 patients required the ICU interventions on admission and during follow-up, respectively. The mean BMI for all patients was 28.4 ± 5.2 kg/m^2^. Fourteen (27.5%) patients presented with normal weight (BMI ≤ 25), 24 (47.1%) with overweight (25 < BMI < 30), and 13 (25.5%) with obesity (BMI ≥ 30; Table 1). The mean duration from onset of clinical signs to admission was identical among BMI groups (7 days; range 2–16; p = 0.283). Patients who required ICU admission presented with a higher BMI than non-ICU patients (29.3 ± 0.80 versus 25.8 ± 1.28; p = 0.048); only one patient with BMI≥30 did not require ICU care. Patients with BMI ≥ 30 tended to be younger than those with normal weight (BMI ≤ 25; 64 versus 58 years; p trend = 0.069). Six (46.2%) patients with BMI ≥ 30 died during hospitalization and these patients were significantly younger than those who died and had BMI≤25 (60 (+/- 15) versus 78 (+/- 9) years, p = 0.041). Hypertension and diabetes were the most common comorbidities (54.9% and 27.5%, respectively, Table 1). Diabetes, chronic obstructive pulmonary disease, and cardiac disease other than arterial hypertension were mainly observed in patients with BMI ≥ 30 (p = 0.024, p = 0.032, and p = 0.029, respectively, Table 1). When removing COPD patients from the analysis, the WHO scale remains correlated with BMI (p = 0.033, S2 Table). No difference for the plasmatic level of C-reactive protein, D-Dimers, troponin, and ferritin was observed at admission between BMI groups. Neutrophils to lymphocytes and CD4 to CD8 ratios did not differ between the BMI groups (S3 Table).

### Immune profiles and BMI categories

**Fig 1:**
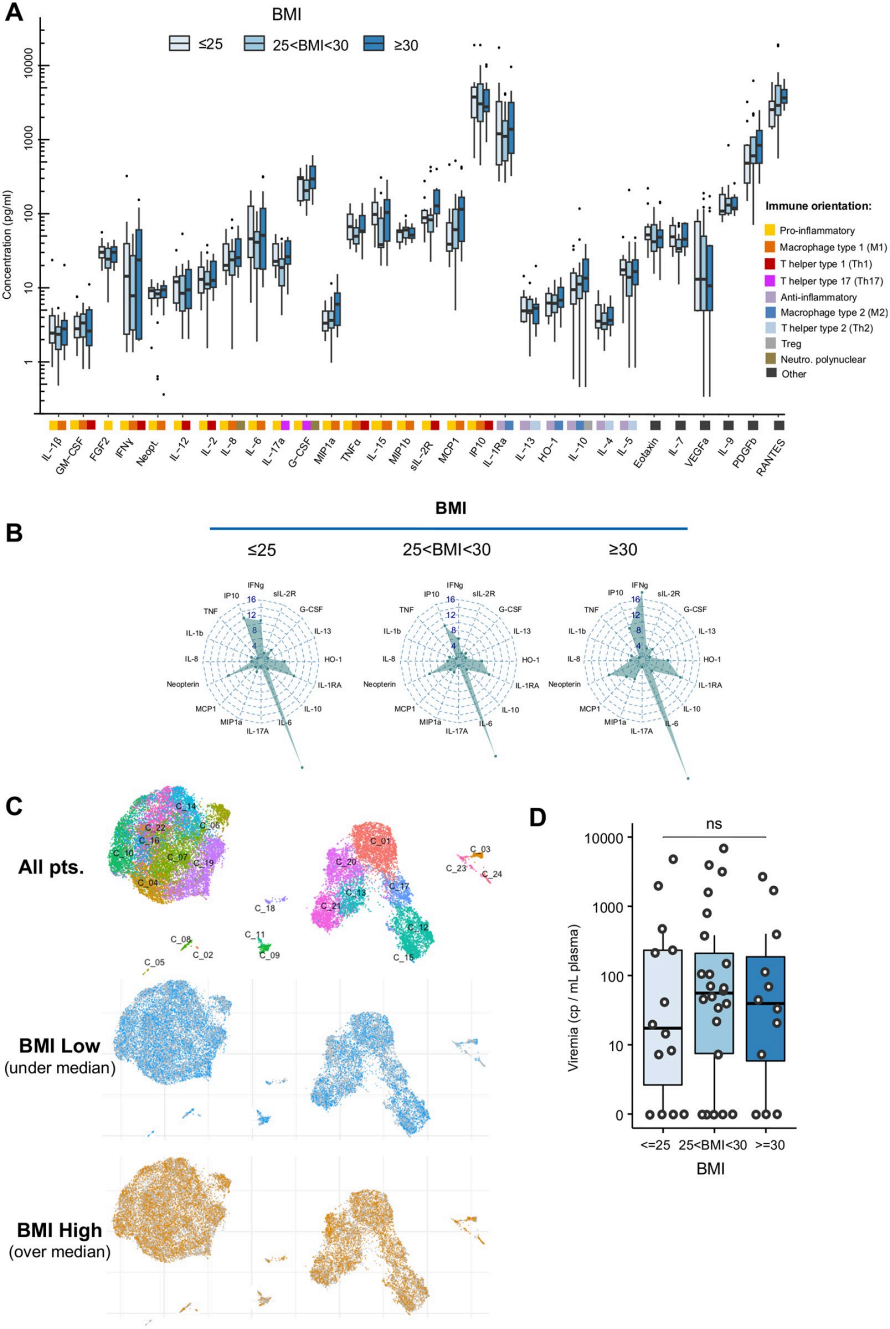
(A) Box plots illustrating the concentration of cytokines according to the BMI levels in COVID-19 patients with BMI ≤ 25, 25 < BMI < 30, and BMI ≥ 30. The colored squares associated with each cytokine illustrate the different orientations of the immune response (n = 42 patients). (B) Radar plots illustrating the fold increase of selected cytokines in patients with BMI ≤ 25 (n = 11), 25 < BMI < 30 (n = 19), and BMI ≥ 30 (n = 12), as compared to healthy donors (n = 18). (C) Characterization of peripheral blood mononuclear cells according to the BMI categories (above and below the median BMI of 26.8, n = 29) using polychromatic flow cytometry and Uniform Manifold Approximation and Projection representation of the 24 clusters identified. Each C stands for one of the 24 « Cluster » automatically identified and defined by the software. Each cluster corresponds to a group of cells with comparable phenotype i.e that express similar levels of the different markers (at their surface or intracellular) (D) Box plots comparing the SARS-CoV-2 RNAaemia measured at the zenith of the cytokines levels, in patients (n = 50) according to the BMI levels (BMI ≤ 25, 25 < BMI < 30, and BMI ≥ 30). Wilcoxon-Mann-Whitney test used for pairwise comparisons in Fig 1D followed by the Benjamini Hochberg test for multiple testing correction in Fig 1A.

On admission, 19 of 30 analyzed cytokines/chemokines or immune-related molecules were significantly increased in blood of patients with COVID-19 compared to controls (all p adjusted = 0.05 to < 0.001, S4 Table). Thirteen of these correlated with the severity of respiratory distress, as evaluated by the WHO scale (S1 Fig). The immune landscape combined nine pro-inflammatory cytokines (e.g. TNFα, IL-8, IFNγ, MCP-1, MIP1α, and G-CSF) together with T helper type 2 or macrophage type 2 cytokines with anti-inflammatory properties (i.e. IL-10, IL-4 and HO-1; S1 Fig). The cytokine levels on admission were not influenced by sex except for IL-15 (S2 Fig), nor by BMI assessed as a continuous variable (S5 Table) or by category (BMI ≤ 25, 25 < BMI < 30, or BMI ≥ 30; Fig 1A, HSI<30 or >36, S3 Fig).

Strong similarity for the immune orientations (e.g. pro versus anti-inflammatory) was observed in patients according to BMI categories (Fig 1B). No difference was observed at the zenith for each cytokine measured from day 1 to day 14 between patients groups classified according to BMI (S3 Fig). Of note, patients receiving corticosteroids at admission (n = 5) had cytokine levels similar to other patients (p>0.05 for each, Wilcoxon test, S4 Fig). Cytokine assays post-therapy anti-IL6R were excluded from analysis. Characterization of peripheral blood mononuclear cells according to BMI categories using polychromatic flow cytometry showed no difference for percentages and absolute numbers of total T cells, CD4 T lymphocytes, CD8 T lymphocytes, B lymphocytes, and NK-T cells (S5 Fig). A decrease trend for the absolute number of NK cells and subsets of monocytes was observed for increasing BMI categories (S5A Fig). Uniform Manifold Approximation and Projection representation of the immune cell landscape (Fig 1C) revealed very similar patterns between BMI subgroups with no significant difference among clusters (S5B Fig). RNAaemia measured at the zenith was identical among BMI groups (Fig 1D). Altogether, the severity of COVID-19 in patients with obesity was neither associated with an increased production or a distinct pattern of cytokines or immune cells nor with a higher SARS-COV2 viremia.

### Relationship between cytokine profiles and the COVID-19 severity according to BMI

**Fig 2:**
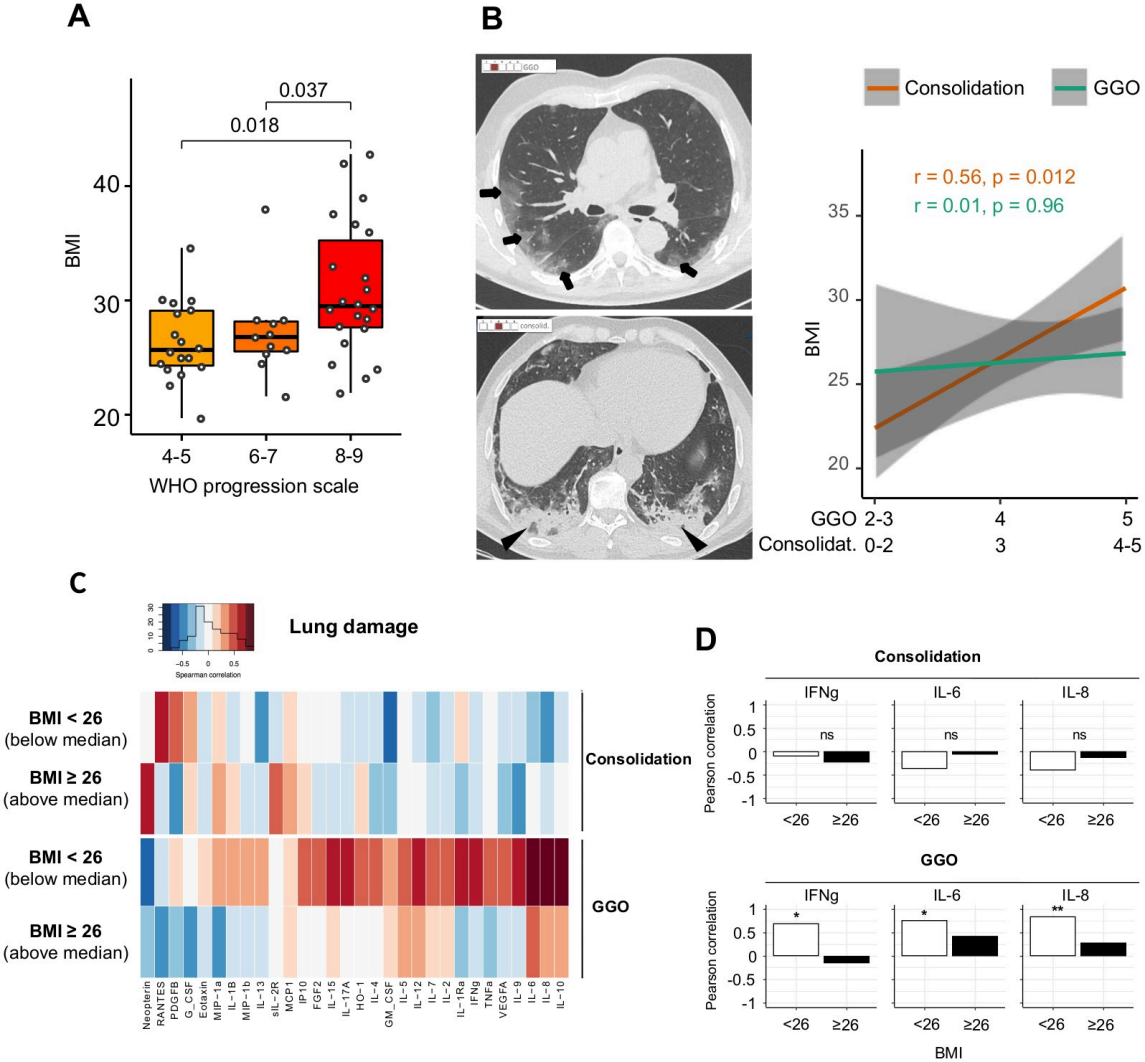
(A) Left part: box plots illustrating BMI in COVID-19 patients (n = 50) according to the level of respiratory distress (the WHO scale). Wilcoxon-Mann-Whitney test used for pairwise comparisons. (B) Left part: High-resolution axial computed tomography (CT) images of two patients show ground glass opacity (GGO; arrows) with a low score #2 and consolidation (arrowheads) with a moderate score #3. Right part: The correlations between the consolidation scale, GGO scale, and BMI. R for the Kendall rank correlation coefficient. (C) The Spearman correlation heatmap depicting the negative (blue), positive (red), and no (white) correlation between consolidation or the GGO extent evaluated based on chest CT images and the blood cytokines levels in COVID-19 patients (n = 19) according BMI below or above 26, i.e. the median BMI of patients with available CT (D) Bar plots showing the Spearman rank correlation coefficient of IFNγ, IL-6, and IL-8 with consolidation (top) or the GGO extent (bottom) according to the BMI levels (n = 19; *p < 0.05; **p < 0.01).

The severity of respiratory distress correlated with the BMI levels (Fig 2A).

Patients with mechanical ventilation and pO2/FIO2 < 150, spO2/FIO2 < 200, or vasopressors (WHO score 8), and vasopressors, dialysis, or extracorporeal membrane oxygenation (WHO score 9) presented with a significantly higher BMI as compared to those with 6–7 WHO score (i.e. requiring oxygen by NIV or high flow or mechanical ventilation and pO2 /FIO2 > 150 or spO2/FIO2 > 200; p = 0.037) and those without oxygen therapy, oxygen by mask, or nasal prongs (4–5 WHO score; p = 0.018). If we remove patients presenting with COPD from the analysis, the correlation between BMI and WHO progression scale remains significant (p trend = 0.033, S2 Table).

The BMI was correlated with the extent of consolidation (r = 0.56; p = 0.012), but not with GGO (r = 0.01; p = 0.96; Fig 2B) as assessed by CT when this examination performed at admission was available (n = 19). No association between the cytokine’s levels and the extent of consolidation and GGO was observed in patients with a high BMI (Fig 2C and 2D). Contrarily, a positive correlation was observed between the extent of GGO and cytokines involved in the Th1 immune orientation (IFNγ, IL-12) or inflammation (IL-6, IL-8, TNFα, IL-17a) in patients with low BMI (Fig 2D). Thus, pulmonary damages in BMI-high patients are distinguished by a higher propensity to make condensation and a lesser influence of cytokines.

**Fig 3:**
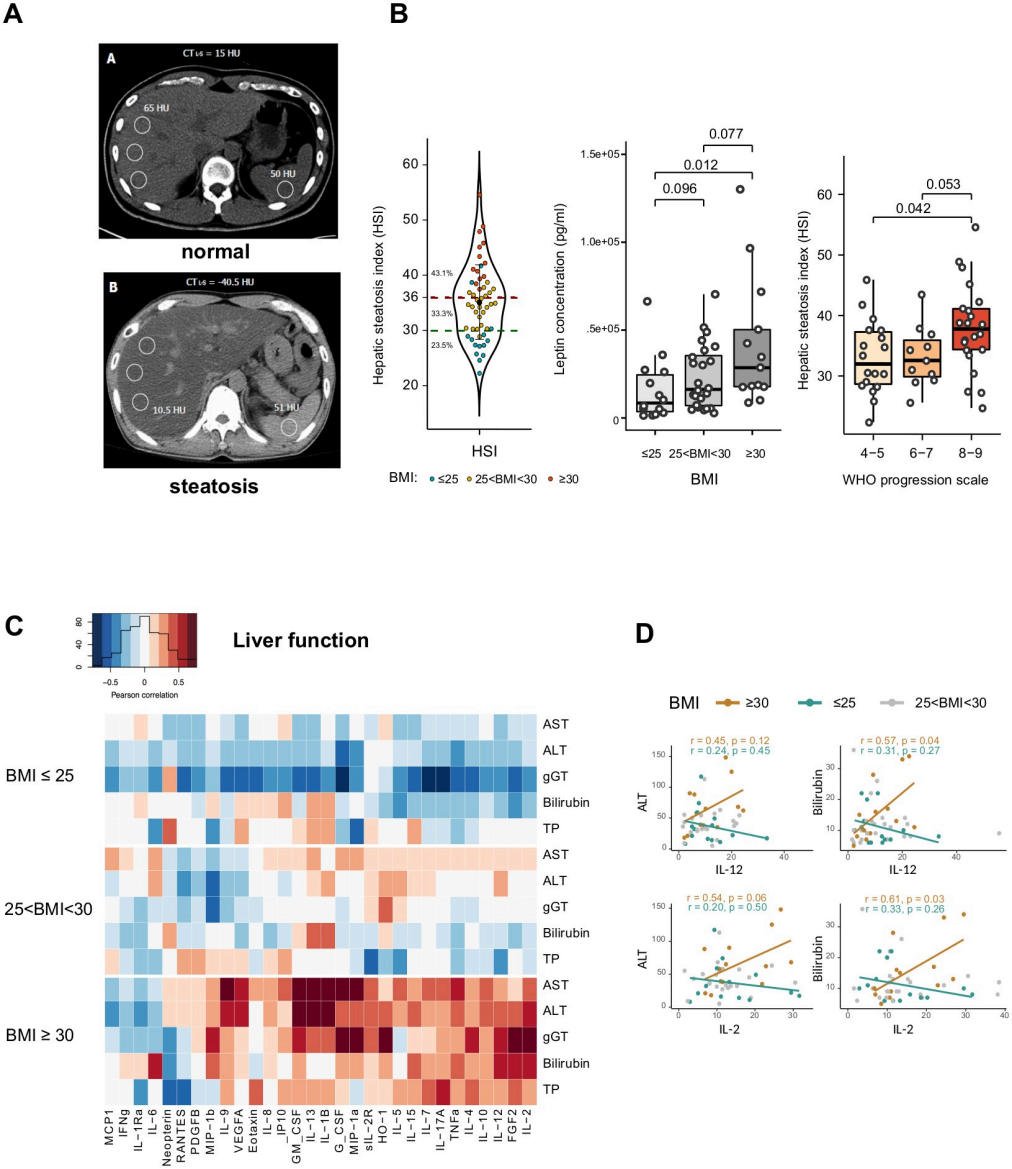
(A) Axial computed tomography images showing normal (up) and low attenuation (down) values of the liver parenchyma reflecting steatosis (n = 23). (B) Left panel: violin plot representing the biological hepatic steatosis index (HSI) according to BMI (≤ 25 in green, 25 < BMI < 30 in yellow, ≥ 30 in red). Middle panel: Box plots depicting the plasma leptin levels of patients (n = 51) according to BMI (BMI ≤ 25, 25 < BMI < 30, ≥ 30). Wilcoxon-Mann-Whitney test used for pairwise comparisons Left panel: Box plots representing the HSI levels according to severity of pulmonary distress (the WHO scale). (C) The Pearson correlation heatmap depicts the negative (blue), positive (red), and no (white) correlation between markers of liver dysfunction (AST, ALT, gGT, TBIL, and PT) and the blood cytokines levels in in patients (n = 51) according to BMI (BMI ≤ 25, 25 < BMI < 30, BMI ≥ 30). (D) Linear regression curves estimated between ALT, total bilirubin, and plasma concentration of IL-1β, IL-12 and IL-2 in non-overweight patients (BMI ≤ 25, in light blue) and in patients with obesity (BMI ≥ 30, in dark blue). Patients with 25 < BMI < 30 are presented in grey.

Obesity is a positive risk factor for NAFLD. Computed tomography identified nine (out of 23; 39.1%) COVID-19 patients with CT_L-S_ attenuation values reflecting hepatic steatosis (Fig 3A) and 12 (out of 23; 52.2%) patients with liver dimensions compatible with hepatomegaly.

In accordance, the biological HSI > 36, reflecting NAFLD, was observed in 43.1% of patients (22/51; Fig 3B) and leptin, produced by fat cells, was increased in blood of patients with obesity (p = 0.012; Fig 3B). The severity of ARDS correlated with HSI (the WHO scale: 8–9 versus 4–5 p = 0.042; Fig 3B), as previously observed with BMI. At admission, 62.7% (n = 32/51) of patients presented with mild liver dysfunction (i.e. abnormalities in ALT, AST, TBIL, and/or GGT; S1 Table). No difference of cytokines levels was observed between patients with low (<30) and high (>36) HIS (S6 Fig). However, of all patients, a significant positive correlation between liver function tests at admission and 5/7 inflammatory cytokines, 6/11 M1 cytokines, and 4/7 Th1 cytokines was observed only in those with obesity (Fig 3C). Correlation plots of IL12 and IL-2 with ALT and TBIL are illustrated in Fig 3D. Altogether, COVID-19 patients with obesity frequently had imaging and biological signs of steatosis and a correlation was observed between liver dysfunction and inflammatory cytokines.

**Fig 4:**
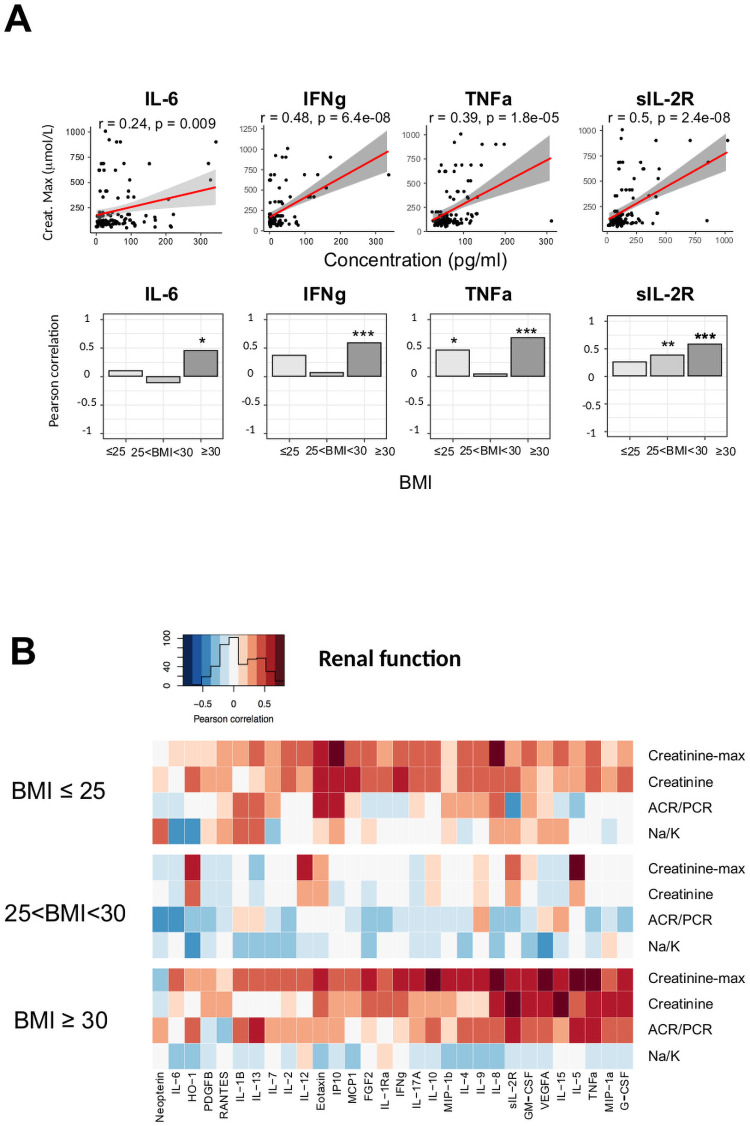
(A) Top panel: Linear regression curves between plasma concentration of IL-6, IFNγ, TNFα, GM-CSF, and the maximum level of creatinine. Bottom panel: bar plots showing the coefficient of the Spearman correlation of IL-6, IFNγ, TNFα, and GM-CSF with the maximum level of creatinine according to BMI (BMI ≤ 25, 25 < BMI < 30, BMI ≥ 30; *p < 0.05; **p < 0.01, ***p < 0.001). (B) The Spearman correlation heatmap depicts the negative (blue), positive (red), and no (white) correlation between markers of kidney dysfunction (i.e. maximum creatinine, creatinine, the albumin/creatinine ratio with protein/creatinine, the Na/K ratio, and the blood cytokines levels according to BMI (BMI ≤ 25, 25 < BMI < 30, BMI ≥ 30).

Overall, 58% of the total cohort (30/51 patients), showed kidney function impairment with increased serum creatinine on admission or during hospitalization (S3 Table). Peak level of serum creatinine during follow-up was higher in patients with BMI ≥ 30 (267 versus 114 μm/L in patients with BMI ≤ 25; overall p = 0.008, S3 Table). Most of inflammatory cytokines levels at admission correlated with the peak creatinine during hospitalization (e.g. IL-6 [r = 0.24; p = 0.0092], IFNγ [r = 0.48, p < 0.001], TNFα [r = 0.39; p < 0.001], and sIL2R [r = 0.5, p < 0.001]; Fig 4A).

This association was reinforced in patients with obesity (Fig 4A). Nine proinflammatory cytokines compatible with M1 polarization (IL-8, FGF, sIL-2R, GM-CSF, TNFα, MIP1α, G-CSF, IFNγ, and MIP1β) correlated with renal dysfunction (i.e. peak of serum creatinine), but also with the ratio urine albumin/creatinine to protein/creatinine (ACR/PCR), reflecting glomerular involvement (Fig 4B).

### Relationship between cytokine profiles and endothelial biomarkers according to BMI

**Fig 5:**
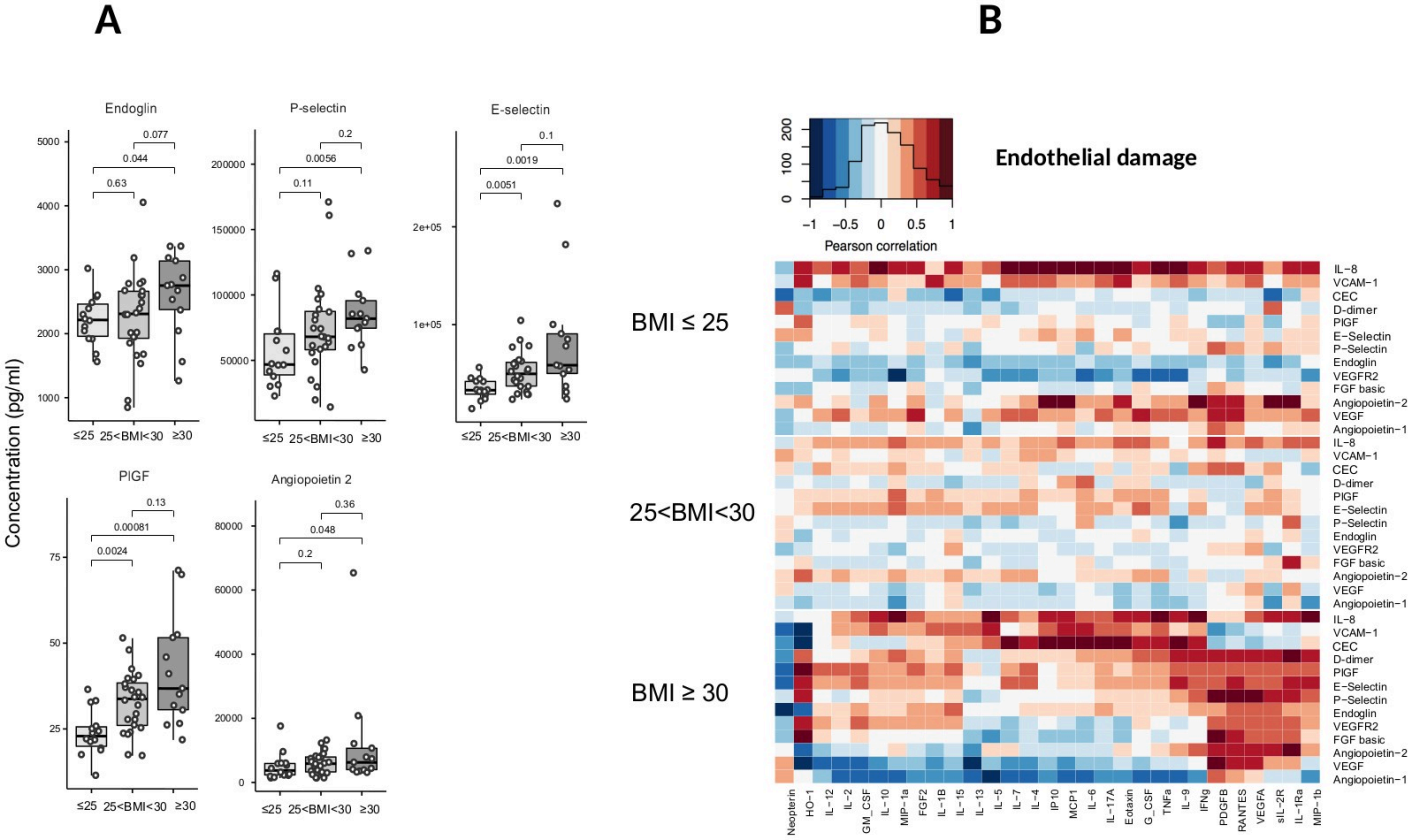
(A) Box plots illustrating the plasma levels of endothelial activation markers (IL-8, endoglin, E and P-selectins, PIGF, Ang-2) according to BMI (BMI ≤ 25, 25 < BMI < 30, BMI ≥ 30). (B) The Spearman correlation heatmap depicts the negative (blue), positive (red), and no (white) correlation between plasma markers of endothelial activation and the blood cytokines levels in COVID-19 patients (n = 38) according to BMI (BMI ≤ 25, 25 < BMI < 30, BMI ≥ 30).

Multiorgan dysfunction in patients with obesity could be related to endothelial damage under cytokine disorders. We measured plasmatic endothelial markers at the zenith of inflammatory cytokines cumulation. Increased levels of endoglin, E and P-selectin, angiopoietin 2, and PIGF were observed in patients with BMI≥30 (Fig 5A and S3 Table).

Concomitantly, markers of endothelial dysfunction correlated with cytokines levels of the Th1 immune orientation (IFNγ, TNFα), inflammation (IL-17a, MIP1α, G-CSF, TNFα, MIP1β, and IFNγ), and/or M1 activation (IL-6, IFNγ, TNFα, IP10, MIP1α, and MCP1), mostly in patients with obesity (Fig 5B). IL-8, a cytokine also produced by activated endothelial cells, was highly correlated with these inflammatory cytokines.

**Fig 6:**
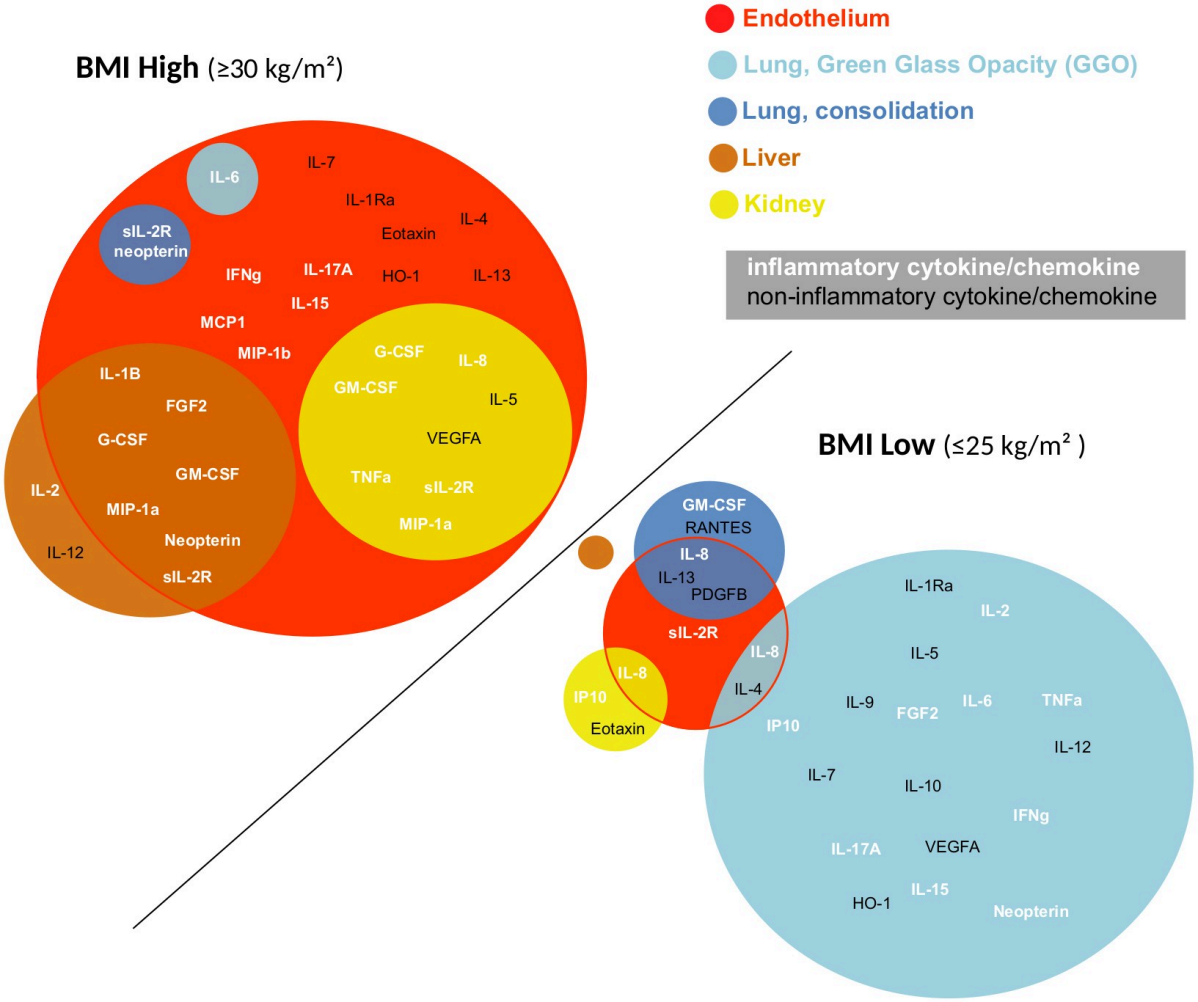
Diagram representing the cytokines correlated with multiorgan damage of endothelial vessels, lung (consolidation and GGO), liver, and kidney in patients with obesity (BMI ≥ 30) as compared to normal-weight patients (BMI ≤ 25). Inflammatory cytokines are illustrated in white and noninflammatory in black.

Overall, a correlation between distinct cytokine patterns and organ dysfunction was revealed in patients with obesity as compare to non-overweight patients (Fig 6).

This interaction did not appear to apply to lung damage. Contrarily, in normal weight patients, Th1/M1/inflammatory cytokines correlated with lung damage (i.e. GGO), with no obvious signs of liver, renal, or endothelial functions damage (Fig 6).

## Discussion

Our study reveals distinct physiopathological mechanism associated with severe COVID-19 in patients with obesity. An association between inflammatory cytokines and liver, renal, and endothelial dysfunctions was revealed in patients with obesity. The severity of lung damage (i.e. consolidation at CT scans) correlated with BMI, but was independent of the cytokine levels. Contrarily, in normal weight patients, Th1/M1/inflammatory cytokines correlated with the early signs of lung damage (GGO), but without any obvious liver, renal, or endothelial dysfunction. This could have significant implications for monitoring and treatment of SARS-COV2 infected patients. We did not observe significant difference between the BMI groups of biomarkers previously reported as reflects of COVID-19 inflammatory immune response such as neutrophils to lymphocytes or CD4 to CD8 ratios [29, 30].

In patients with obesity, overproduction of cytokines evokes a preexisting chronic inflammation, since adipocytes secretion of pro-inflammatory molecules (e.g. MCP-1, IL-6, IL-18, TNF-α, IL-1β) together with a decrease of anti-inflammatory cytokines (e.g. IL-10) has been reported [31]. We did not observe any obvious difference in cytokine overproduction or immune orientation between obese and normal-weight patients in accordance with previous observation [32]. A differential impact of inflammatory cytokines on liver, lung, kidney, and vascular/endothelial functions, involved in disease prognosis [33], was therefore assumed. The incidence of acute respiratory distress syndrome is increased in patients with obesity [34, 35] and the effects of increased inflammatory cytokines are suspected. Here we showed that consolidation at CT scan was clearly independent of the cytokine levels, but positively associated with BMI. Contrarily, a strong correlation was observed between inflammatory cytokines and the extent of GGO in normal-weight patients. This observation suggests distinct pathogenesis of pulmonary lesions in COVID-19 patients and their relationship with systemic inflammation-related disorders. This field is of particular importance since anti-IL-6 receptor treatments, which attempt to reduce hyperinflammation and attenuate lung damage have shown variable levels of the efficacy in COVID19 patients [36–38].

The liver is a potential target for SARS-CoV-2 [39]. Hepatic dysfunction, seen in 14%-53% of patients with SARS-CoV-2 infection, is associated with severe disease [40]. Liver dysfunction could be related to uncontrolled immune reaction, cytopathic effect of the virus, and/or drug induced liver injury. SARS-Cov2 RNAemia was identical in all BMI groups as previously observed [41]. A significant correlation between liver dysfunction (AST, ALAT, TBIL, and prothrombin time) and inflammatory cytokines was solely observed in patients with obesity suggesting a preexisting liver abnormalities/sensitivity to cytokines such as TNFα involved in many forms of liver injury [16].

Acute kidney injury (AKI) has been reported to occur in 36.6% of COVID19 patients and in temporal association with respiratory failure. The mortality rate for AKI is 35% [22]. The causes of kidney involvement could be multifactorial. Besides cardio-renal syndrome, virus particles are observed in renal endothelial cells [42], tubular epithelium, and podocytes through an angiotensin converting enzyme 2-dependent pathway [43], suggesting a direct contribution of viremia to AKI. Furthermore, hyperinflammation, macrophage activation and microthrombi in the context of endotheliitis have been suspected to contribute to AKI. We did not evidence correlation between viremia and the extent of AKI (r = 0.14, p = 0.35), arguing against a significant impact of SARS-CoV-2 viremia on kidney function. We showed an association between many inflammatory cytokines and kidney dysfunction, the greater part of which may be produced by activated type 1 macrophages. This association was strengthened in patients with BMI ≥ 30.

Endothelial dysfunction has been reported as a main physiopathological feature in COVID-19, revealed by different histopathological studies [44, 45] and partially explained by the high endothelial expression of angiotensin converting enzyme 2, the SARS-CoV-2 receptor [46]. Moreover, human obesity is associated with vascular endothelial dysfunction due to oxidative stress, inflammation, and the enzymatic pathways involving perivascular adipose tissue and vasculature [17, 47, 48]. Consistent with these studies, we observed a positive correlation between markers associated with endothelial dysfunction and BMI, suggesting that endotheliitis may occur more frequently in patients with obesity. Accordingly, the levels of numerous inflammatory cytokines were associated with endothelial dysfunction in patients with obesity. It can be hypothesized that patients with obesity are more susceptible to the SARS-CoV-2 infection of endothelial cells and/or have sensitized endothelium more prone to microvascular lesions under inflammatory cytokines. Strikingly, dysfunction of kidney and liver in patients with obesity involved a common subset of cytokines to that observed with endothelial dysfunction, suggesting physiopathological similarities. Hyperinflammation observed in patients without obesity did not correlate with biological signs of endothelial dysfunction, but was strongly associated with the early signs of lung damage, evidenced by the occurrence of GGO on CT scans.

There are limitations to our study. First, the small number of studied patients leading to low statistical power, however, an extensive immune exploration allowed us to confirm the trends observed. Second, all patients presented with a severe form of the disease. A controlled group composed of patients who received home care or of high-risk patients who benefited from an early extensive immunomonitoring before the emergence of ARDS would have provided important information to decipher mechanisms initiating the cytokinic storm. However, our results showing a comparable cytokine landscape and viremia among different BMI groups and a differential impact of hyperinflammation on vital organs functions in patients with obesity, the latter possibly due to a higher endothelial susceptibility, may help to better identify and manage patients who are at higher-risk for severe form of COVID-19.

## Supporting information

S1 Table Description of the antibodies used in flow cytometry experiments. (PDF)

S2 Table Respiratory distress of patients without COPD. (PDF)

S3 Table Biological characteristics. (PDF)

S4 Table Cytokine levels in healthy donors and patients at admission. (PDF)

S5 Table Cytokine levels at admission and correlation to BMI (as continuous variable). (PDF)

S1 Fig Box plots illustrating a significant increase of the cytokines concentration in COVID-19 patients (n = 15) according to the level of respiratory functional status (the WHO scale). The cytokines levels of healthy donors (n = 18) are shown. The colored squares associated with each cytokine illustrate the different orientations of the immune response. (*p<0.05; **p<0.01, ***p<0.001, ****p<0.0001).
(PDF)

S2 Fig Box plots illustrating the concentration of cytokines at admission according to the sex of COVID-19 patients (n = 42). Wilcoxon-MannWhitney tests used for pairwise comparisons followed by the Benjamini Hochberg test for multiple testing correction. * P value <0.05.
(PDF)

S3 Fig Box plots illustrating the zenith (maximum concentration) of each cytokine measured from day 1 to day 14, according to the BMI levels in COVID-19 patients (n = 51). Wilcoxon-Mann-Whitney tests used for pairwise comparisons followed by the Benjamini Hochberg test for multiple testing correction. * Pvalue <0.05.
(PDF)

S4 Fig Box plots illustrating the concentration of cytokines according to corticosteroids administration to COVID-19 patients (n = 51). Wilcoxon Mann-Whitney test used for pairwise comparisons followed by the Benjamini Hochberg test for multiple testing correction.
(PDF)

S5 Fig **(A)**. Percentage and absolute number (mean ± standard deviation) of lymphocytes, B lymphocytes, NK cells, NK-T cells, T lymphocytes, CD4 and CD8 T cell subsets; classical, intermediate and non-classical, CD163 and CD163 monocytes. **(B)** Bar plots representing the percentage of different clusters identified by Excyted pipeline in COVID-19 patients according to the BMI categories (above and below the median BMI of 26.8). Exact p value is reported if significant.
(PDF)

S6 Fig Box plots illustrating the concentration of cytokines according to HSI levels in COVID-19 patients (n = 51). Wilcoxon-Mann-Whitney test used for pairwise comparisons followed by the Benjamini Hochberg test for multiple testing correction.
(PDF)

S7 Fig Box plots illustrating the concentration of cytokines according to the sex, age, arterial hypertension, diabetes, COPD among BMI > 30 COVID-19 patients. Wilcoxon-Mann-Whitney tests used for pairwise comparisons followed by the Benjamini Hochberg test for multiple testing correction.
(PDF)

S1 Data (XLSX)
