# Supplementary material for: Differential association between inflammatory cytokines and multiorgan dysfunction in COVID-19 patients with obesity
Source: PLoS One. 2021 May 26;16(5):e0252026. doi: 10.1371/journal.pone.0252026 (PMC8153504; doi:10.1371/journal.pone.0252026)
Supplement: S1 Table — (PDF) [file pone.0252026.s001.pdf]

S1 Table: Description of the antibodies used in flow cytometry experiments.

Antibody	Fluorochrome	Clone	Provider	Catalog number	Dilution factor
CD3	EDC	UCHT1	Beckman Coulter	A07748	400
CD8	APC	RPA-T8	BD	555369	100
CD14	BV650	M5E2	BD	563419	200
CD16	BV605	3G8	BD	563172	100
CD19	BV450	HIB19	BD	560353	400
CD40	APC-Cy7	5C3	Biolegend	334324	100
CD56	PE-Cy7	B159	BD	557747	100
CD86 (B7-2)	AF700	2331	BD	561124	400
CD163	PerCP-Vio700	REA812	Miltenyi Biotec	130-112-291	100
CD209 (DC-SIGN)	FITC	DCN46	BD	551264	100
CD299 (DC-SIGNR)	PE	-	R et D	FAB162P	100
HLA-DR	BV785	L243	BioLegend	307642	400
Live/Dead	-	-	Life Technologies	L34959	200
