# Supplementary material for: Differential association between inflammatory cytokines and multiorgan dysfunction in COVID-19 patients with obesity
Source: PLoS One. 2021 May 26;16(5):e0252026. doi: 10.1371/journal.pone.0252026 (PMC8153504; doi:10.1371/journal.pone.0252026)
Supplement: S2 Table — (PDF) [file pone.0252026.s002.pdf]

**S2 Table : Respiratory distress of patients without COPD**

| | [ALL] (N=47) | <=25 (N=14) | 25-30 (N=23) | >=30 (N=10) | p overall | p trend |
|---|---|---|---|---|---|---|
| **ICU required on admission** | | | | | 0.492 | 0.212 |
| No | 14 (29.8%) | 6 (42.9%) | 6 (26.1%) | 2 (20.0%) | | |
| yes | 33 (70.2%) | 8 (57.1%) | 17 (73.9%) | 8 (80.0%) | | |
| **ICU required during hospitalization** | | | | | 0.131 | 0.072 |
| No | 8 (17.0%) | 5 (35.7%) | 2 (8.70%) | 1 (10.0%) | | |
| yes | 39 (83.0%) | 9 (64.3%) | 21 (91.3%) | 9 (90.0%) | | |
| **WHO scale#** | | | | | 0.137 | **0.033** |
| 4-5 | 17 (36.2%) | 8 (57.1%) | 7 (30.4%) | 2 (20.0%) | | |
| 6-7 | 11 (23.4%) | 2 (14.3%) | 8 (34.8%) | 1 (10.0%) | | |
| 8-9 | 19 (40.4%) | 4 (28.6%) | 8 (34.8%) | 7 (70.0%) | | |
| **Death** | | | | | 0.114 | 0.356 |
| No | 35 (74.5%) | 8 (57.1%) | 20 (87.0%) | 7 (70.0%) | | |
| yes | 12 (25.5%) | 6 (42.9%) | 3 (13.0%) | 3 (30.0%) | | |

**WHO scale#**

- Without oxygen therapy or oxygen by mask or nasal prongs (score 4-5)
- Oxygen by NIV or high flow or mechanical ventilation and pO2 /FIO2 >150 or spO2/FIO2>200 (score 6-7)
- Mechanical ventilation and pO2/FIO2<150 or spO2/FIO2<200 or vasopressors (score 8), and vasopressors or dialysis or ECMO (score 9)
