# Supplementary material for: Differential association between inflammatory cytokines and multiorgan dysfunction in COVID-19 patients with obesity
Source: PLoS One. 2021 May 26;16(5):e0252026. doi: 10.1371/journal.pone.0252026 (PMC8153504; doi:10.1371/journal.pone.0252026)
Supplement: S3 Table — (PDF) [file pone.0252026.s003.pdf]

S3 Table : Biological characteristics

	Body Mass Index					
	[ALL] N=51	≤25 N=14	25 < BMI <30 N=24	≥30 N=13	p overall	p trend
**Respiratory functional status**						
**WHO scale**#					0.103	**0.029**
4-5	18 (35.3%)	8 (57.1%)	7 (29.2%)	3 (23.1%)		
6-7	11 (21.6%)	2 (14.3%)	8 (33.3%)	1 (7.69%)		
8-9	22 (43.1%)	4 (28.6%)	9 (37.5%)	9 (69.2%)		
**Liver function**						
AST (UI/L)	57.0 [40.0;73.5]	53.5 [35.5;63.8]	54.5 [36.5;77.0]	58.0 [42.0;81.0]	0.432	0.216
ALT (UI/L)	39.0 [28.0;58.5]	27.0 [16.2;46.2]	42.0 [31.8;54.2]	62.0 [38.0;88.0]	0.034	0.009
gGT (UI/L)	94.0 [55.0;147]	56.0 [24.5;98.0]	110 [84.5;152]	112 [73.5;329]	0.179	0.089
Total Bilirubin (µmol/L)	10.0 [8.00;14.0]	9.00 [7.00;12.5]	10.0 [8.00;13.0]	13.0 [9.00;17.0]	0.273	0.113
PT (%)	85.0 [79.0;92.2]	88.0 [75.0;93.0]	85.0 [79.8;96.2]	81.0 [75.0;88.0]	0.594	0.432
HSI	34.9 [30.4;38.8]	27.9 [26.2;29.3]	34.6 [32.2;36.4]	42.3 [39.9;45.9]	**<0.001**	**<0.001**
**Renal function**						
Max. Creatinine (µmol/L)	117 [90.0;192]	114 [97.2;160]	98.0 [70.0;124]	267 [142;564]	**0.008**	0.147
Creatinine (µmol/L)	85.0 [67.2;122]	88.5 [77.0;101]	74.0 [59.0;106]	110 [84.0;195]	0.087	0.465
ACR/PCR (%)	13.4 [7.69;29.5]	15.5 [7.29;37.1]	13.2 [7.75;29.8]	11.4 [9.56;19.4]	0.812	0.542
Na/K	0.55 [0.33;1.31]	0.98 [0.38;2.12]	0.51 [0.34;1.03]	0.60 [0.29;0.76]	0.505	0.323
**Blood vessels**						
Angiopoietin 1* (pg/ml)	10852 [4742;25115]	11144 [6239;22050]	12860 [5055;22288]	8891 [3953;34327]	0.967	0.927
Angiopoietin 2* (pg/ml)	5323 [3115;7863]	3737 [2209;5919]	5853 [3132;7929]	6251 [3847;10644]	0.132	**0.044**
CECs per mL	22.5 [10.8;51.5]	75.5 [41.0;112]	11.5 [9.25;28.5]	22.5 [18.5;43.0]	0.203	0.371
D-dimer* (ng/ml)	2447 [988;4275]	1126 [836;2882]	1504 [1005;3978]	5246 [3052;5928]	0.096	**0.047**
E-selectin* (pg/ml)	45406 [32176;60505]	33020 [28504;41978]	49787 [36815;61465]	58571 [49730;90663]	**0.002**	**<0.001**
Endoglin* (pg/ml)	2369 [1946;2711]	2215 [1951;2461]	2309 [1922;2664]	2751 [2369;3136]	0.093	**0.042**
FGF-basic* (pg/ml)	26.2 [17.4;48.2]	20.3 [13.5;22.9]	31.8 [17.5;59.1]	29.8 [21.7;61.8]	0.166	0.099
IL-8* (pg/ml)	30.8 [19.1;52.1]	30.3 [14.3;43.9]	27.3 [21.7;41.9]	53.0 [22.5;71.7]	0.161	0.062
P-selectin* (pg/ml)	68209 [47975;86152]	46817 [38615;70289]	67981 [57776;87375]	81909 [74337;95647]	0.027	0.006
PlGF* (pg/ml)	31.8 [23.9;37.6]	22.9 [19.8;25.6]	33.8 [25.9;38.4]	36.8 [30.4;51.6]	**0.001**	**<0.001**
VCAM1* (ng/ml)	3065.2 [1841.2;469.7]	4486.9 [1954.9;6380.6]	2339.6 [1556.5;3739.1]	3475.1 [2230.6;5610.8]	0.200	0.997
VEGF* (pg/ml)	98.1 [19.6;184]	85.5 [37.0;139]	121 [31.5;216]	86.4 [12.5;158]	0.738	0.784
VEGFR2* (pg/ml)	10422 [8064;12305]	11679 [9503;14020]	9152 [7015;11558]	11219 [8543;12743]	0.156	0.546
**Biology at admission**						
CRP (mg/L)	186 [127;240]	132 [112;167]	202 [162;296]	186 [174;210]	**0.025**	0.060
Ferritin (µg/L)	1298 [719;1874]	1074 [470;1375]	1464 [1036;2339]	1208 [621;1465]	0.222	0.729
D-dimer (ng/ml)	1657 [1016;3800]	1265 [822;2941]	1438 [1117;4709]	1797 [1336;3476]	0.352	0.191
Troponin (ng/ml)	16.4 [8.27;64.5]	26.9 [20.4;63.0]	11.9 [8.80;18.4]	13.8 [6.83;89.2]	0.316	0.294
Leucocytes (Nb/L)	8.70 [6.20;11.4]	7.75 [5.78;11.0]	9.95 [6.65;13.5]	8.30 [6.20;9.30]	0.218	0.982
PNN (Nb/L)	7.50 [4.89;9.89]	6.61 [3.72;9.52]	8.08 [5.46;12.3]	7.30 [4.93;7.89]	0.310	0.898
PNE (Nb/L)	0.01 [0.00;0.08]	0.00 [0.00;0.10]	0.01 [0.00;0.08]	0.00 [0.00;0.04]	0.422	0.944
PNB (Nb/L)	0.01 [0.00;0.02]	0.01 [0.00;0.02]	0.02 [0.00;0.03]	0.01 [0.00;0.01]	0.157	0.609
Lympho (Nb/L)	0.82 [0.63;1.11]	0.82 [0.64;1.13]	0.90 [0.63;1.15]	0.78 [0.58;0.93]	0.587	0.624
PNL ratio	9.15 [4.99;13.8]	9.27 [3.06;13.9]	9.07 [5.21;13.8]	9.15 [5.30;12.6]	0.929	0.777
Monocytes (Nb/L)	0.46 [0.29;0.62]	0.45 [0.29;0.63]	0.51 [0.34;0.72]	0.35 [0.27;0.46]	0.310	0.404
B cells (Nb/mm^3)	143 [87.5;194]	99.5 [75.0;185]	124 [83.5;210]	154 [116;193]	0.359	0.154
T cells (Nb/mm^3)	499 [370;672]	424 [286;551]	562 [392;716]	530 [401;576]	0.282	0.306
CD4+ T cells (Nb/mm^3)	343 [257;448]	327 [191;397]	354 [289;466]	332 [296;452]	0.544	0.551
CD8+ T cells (Nb/mm^3)	157 [77.0;241]	105 [64.0;154]	160 [93.5;339]	160 [109;203]	0.202	0.317
CD4/CD8	2.52 [1.39;3.85]	2.92 [2.43;4.08]	2.01 [1.15;3.72]	2.37 [1.41;3.71]	0.492	0.484
NK cells (Nb/mm^3)	76.0 [45.5;127]	83.5 [47.5;152]	79.5 [55.8;153]	47.0 [40.0;78.0]	0.100	0.130
Platelets (Nb/L)	237 [174;318]	196 [168;263]	276 [206;335]	223 [164;263]	0.084	0.883
Viremia	43.6 [7.35;231]	17.3 [1.84;231]	55.6 [7.35;209]	39.4 [5.51;186]	0.766	0.785

Continuous variables are expressed as median [IQR]

ACR/PCR = ratio of albuminuria/creatininuria with proteinuria/creatininuria

AST = Asparate aminotransferase

ALT = Alanine aminotransferase

gGT = Gamma glutamyl transpeptidase

TBIL = total bilirubin

PT= Prothrombin time

* = measured at zenith of inflammatory cytokines

WHO scale#

- Without oxygen therapy or oxygen by mask or nasal prongs (score 4-5)
- Oxygen by NIV or high flow or mechanical ventilation and pO2 /FIO2 >150 or spO2/FIO2>200 (score 6-7)
- Mechanical ventilation and pO2/FIO2<150 or spO2/FIO2<200 or vasopressors (score 8), and vasopressors or dialysis or ECMO (score 9)
