# Supplementary material for: Differential association between inflammatory cytokines and multiorgan dysfunction in COVID-19 patients with obesity
Source: PLoS One. 2021 May 26;16(5):e0252026. doi: 10.1371/journal.pone.0252026 (PMC8153504; doi:10.1371/journal.pone.0252026)
Supplement: S4 Table — (PDF) [file pone.0252026.s004.pdf]

S4 Table: Cytokine levels in healthy donors and patients at admission.

	Healthy donors (N=18)	Patients (N=42)	p. value	p. adj
Eotaxin	57.4 (24.8)	62.1 (43.1)	0.854	0.854
FGF2	23.8 (7.24)	29.1 (10.9)	0.061	0.092
G-CSF	73.5 (18.1)	273 (134)	<0.001	<0.001
GM-CSF	1.99 (1.03)	3.53 (2.20)	0.004	0.008
HO1	1.57 (0.31)	6.99 (3.16)	<0.001	<0.001
IFNg	2.15 (3.20)	33.5 (59.7)	<0.001	<0.001
IL-10	3.89 (3.48)	16.7 (19.8)	<0.001	<0.001
IL-12	8.23 (5.11)	12.3 (10.00)	0.170	0.232
IL-13	2.61 (0.89)	6.02 (3.54)	<0.001	<0.001
IL-15	72.9 (47.9)	97.3 (73.8)	0.497	0.552
IL-17A	17.2 (5.82)	25.3 (12.9)	0.016	0.027
IL-1B	0.94 (0.54)	3.51 (4.50)	<0.001	<0.001
IL1-RA	139 (77.1)	2269 (3171)	<0.001	<0.001
IL-2	9.56 (3.97)	14.9 (8.76)	0.043	0.068
IL-4	2.99 (0.79)	4.24 (1.89)	0.016	0.027
IL-5	10.4 (8.60)	22.7 (33.3)	0.011	0.020
IL-6	1.76 (0.61)	70.4 (77.8)	<0.001	<0.001
IL-7	40.3 (11.5)	47.9 (20.0)	0.313	0.376
IL-8	7.97 (2.52)	30.1 (18.5)	<0.001	<0.001
IL-9	139 (21.8)	159 (121)	0.611	0.655
IP10	353 (182)	4621 (4290)	<0.001	<0.001
MCP1	20.2 (8.03)	111 (130)	<0.001	<0.001
MIP1a	1.11 (0.41)	4.91 (3.10)	<0.001	<0.001
MIP1b	61.4 (7.65)	58.5 (13.7)	0.269	0.351
Neopterin	1.31 (1.01)	8.32 (3.38)	<0.001	<0.001
PDGFB	793 (575)	1040 (1134)	0.834	0.854
RANTES	4458 (2492)	4207 (3825)	0.349	0.403
sIL-2R	40.1 (11.0)	126 (98.9)	<0.001	<0.001
TNF	54.5 (9.33)	66.8 (29.7)	0.294	0.368
VEGFA	14.5 (20.5)	42.0 (56.8)	0.141	0.201

Cytokines levels are expressed as mean ± sd
