# Supplementary material for: Differential association between inflammatory cytokines and multiorgan dysfunction in COVID-19 patients with obesity
Source: PLoS One. 2021 May 26;16(5):e0252026. doi: 10.1371/journal.pone.0252026 (PMC8153504; doi:10.1371/journal.pone.0252026)
Supplement: S5 Table — (PDF) [file pone.0252026.s005.pdf]

S5 Table: Cytokine levels at admission and correlation to BMI (as continuous variable).

	correlation to BMI	
	r Kendall	p value
Eotaxin	-0.10	0.357
FGF2	-0.04	0.729
G-CSF	0.10	0.329
GM-CSF	-0.06	0.573
HO1	0.08	0.474
IFNg	0.04	0.719
IL-10	0.07	0.508
IL-12	-0.01	0.957
IL-13	-0.02	0.845
IL-15	-0.08	0.450
IL-17A	0.00	0.965
IL-1B	-0.03	0.770
IL1-RA	0.00	0.965
IL-2	0.05	0.672
IL-4	0.04	0.729
IL-5	-0.09	0.409
IL-6	0.02	0.871
IL-7	-0.01	0.922
IL-8	0.10	0.374
IL-9	0.10	0.363
IP10	-0.10	0.357
MCP1	0.09	0.392
MIP1a	0.12	0.260
MIP1b	0.06	0.588
Neopterin	0.02	0.875
PDGFB	0.09	0.429
RANTES	0.19	0.070
sIL-2R*	0.22	0.042
TNF	0.03	0.770
VEGFA	-0.08	0.491

*p=0.965 after adjutement for multiple testing
