# Supplementary material for: Differential association between inflammatory cytokines and multiorgan dysfunction in COVID-19 patients with obesity
Source: PLoS One. 2021 May 26;16(5):e0252026. doi: 10.1371/journal.pone.0252026 (PMC8153504; doi:10.1371/journal.pone.0252026)
Supplement: S1 Fig — The cytokines levels of healthy donors (n = 18) are shown. The colored squares associated with each cytokine illustrate the different orientations of the immune response. (*p<0.05; **p<0.01, ***p<0.001, ****p<0.0001). (PDF) [file pone.0252026.s006.pdf]

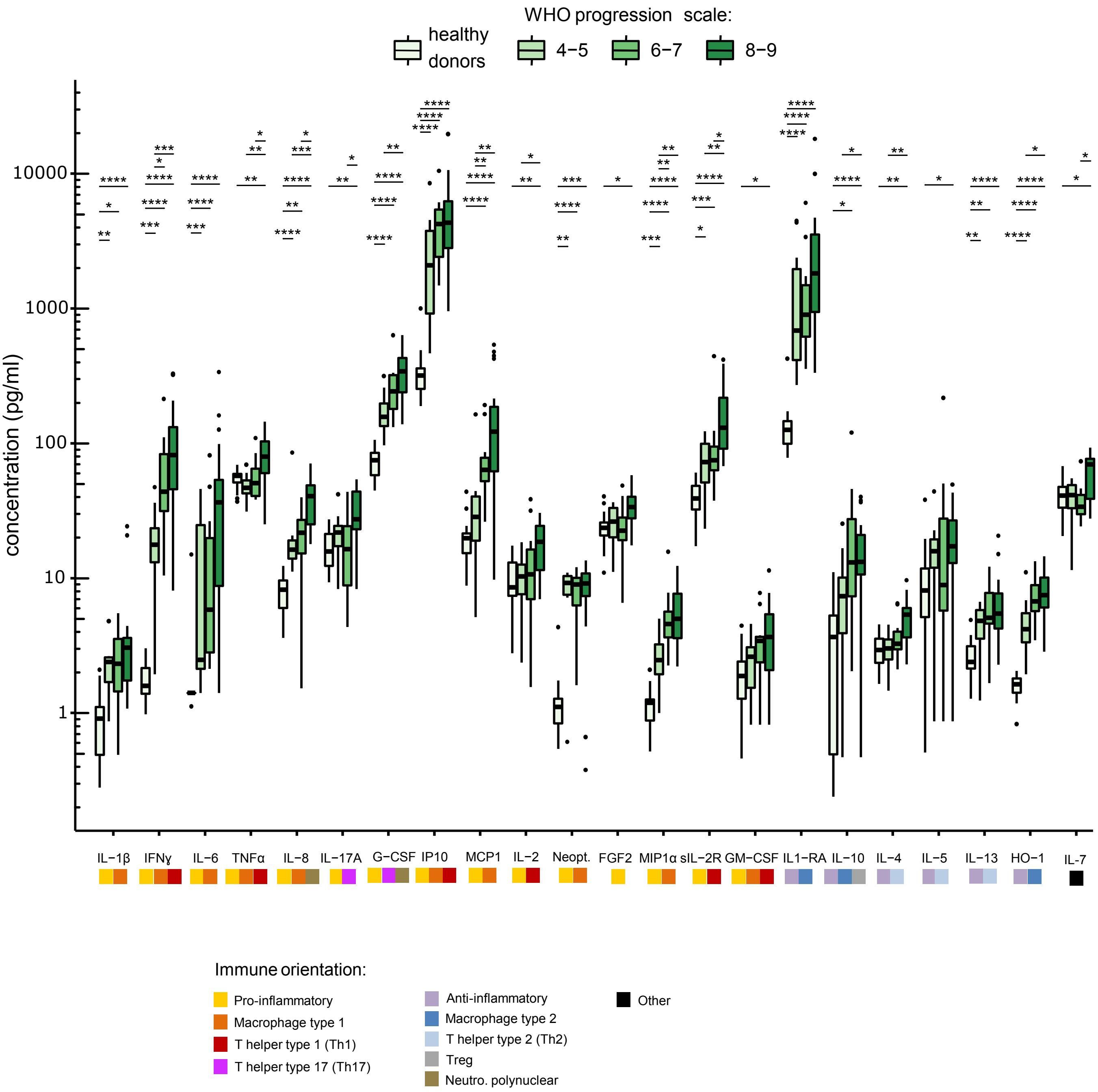

S1 Fig. Box plots illustrating a significant increase of the cytokines concentration in COVID-19 patients (n = 51) according to the level of respiratory functional status (the WHO scale). The cytokines levels of healthy donors (n = 18) are shown. The colored squares associated with each cytokine illustrate the different orientations of the immune response.
(*p<0.05; **p<0.01, ***p<0.001, ****p<0.0001).
