# Supplementary material for: Differential association between inflammatory cytokines and multiorgan dysfunction in COVID-19 patients with obesity
Source: PLoS One. 2021 May 26;16(5):e0252026. doi: 10.1371/journal.pone.0252026 (PMC8153504; doi:10.1371/journal.pone.0252026)
Supplement: S3 Fig — Wilcoxon-Mann-Whitney tests used for pairwise comparisons followed by the Benjamini Hochberg test for multiple testing correction. * Pvalue <0.05. (PDF) [file pone.0252026.s008.pdf]

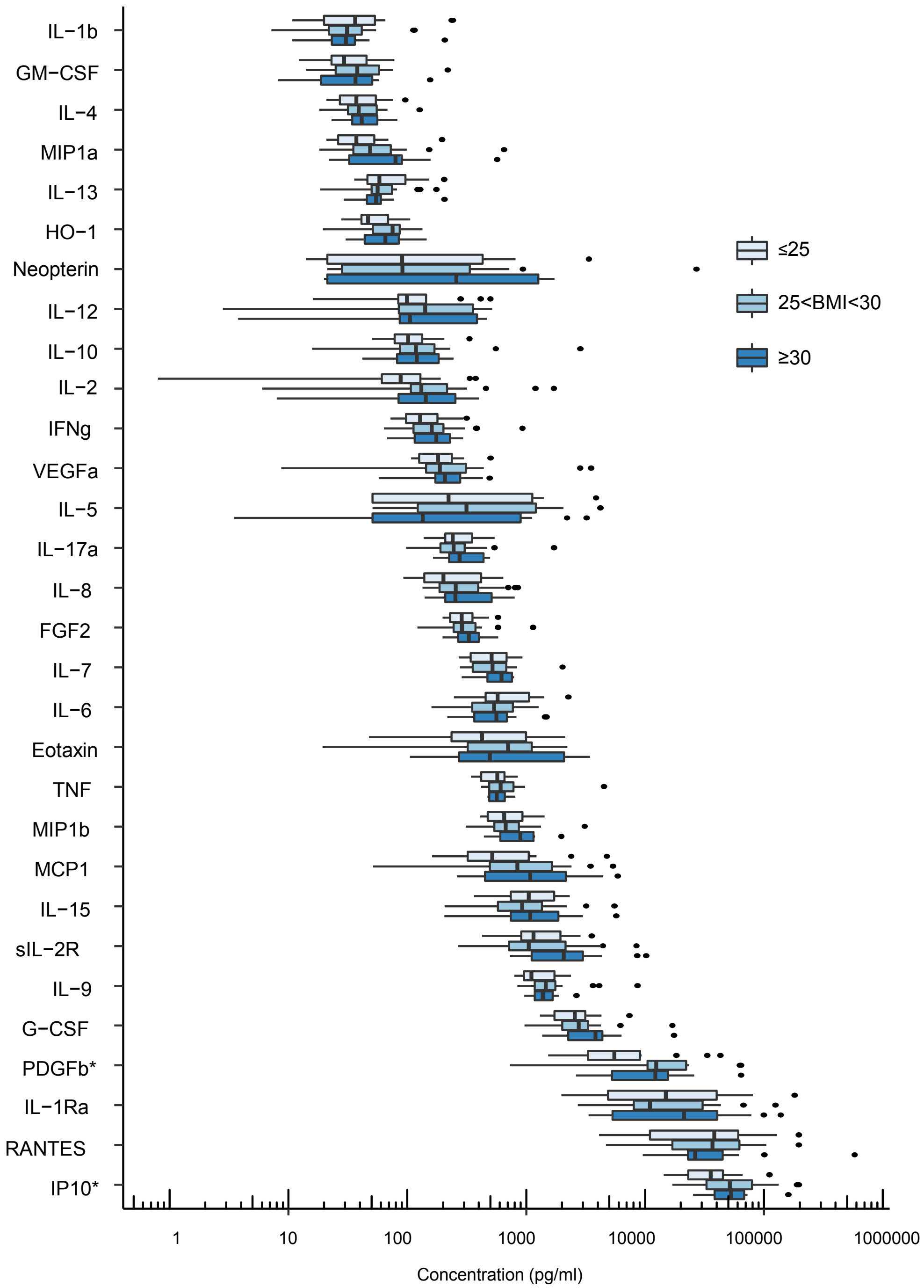

S3 Fig. Box plots illustrating the zenith (maximum concentration) of each cytokine measured from day 1 to day 14, according to the BMI levels in COVID-19 patients (n=51). Wilcoxon-Mann-Whitney tests used for pairwise comparisons followed by the Benjamini Hochberg test for multiple testing correction. * *P* value <0.05
