# Supplementary material for: Differential association between inflammatory cytokines and multiorgan dysfunction in COVID-19 patients with obesity
Source: PLoS One. 2021 May 26;16(5):e0252026. doi: 10.1371/journal.pone.0252026 (PMC8153504; doi:10.1371/journal.pone.0252026)
Supplement: S5 Fig — (A). Percentage and absolute number (mean ± standard deviation) of lymphocytes, B lymphocytes, NK cells, NK-T cells, T lymphocytes, CD4 and CD8 T cell subsets; classical, intermediate and non-classical, CD163 and CD163 monocytes. (B) Bar plots representing the percentage of different clusters identified by Excyted pipeline in COVID-19 patients according to the BMI categories (above and below the median BMI of 26.8). Exact p value is reported if significant. (PDF) [file pone.0252026.s010.pdf]

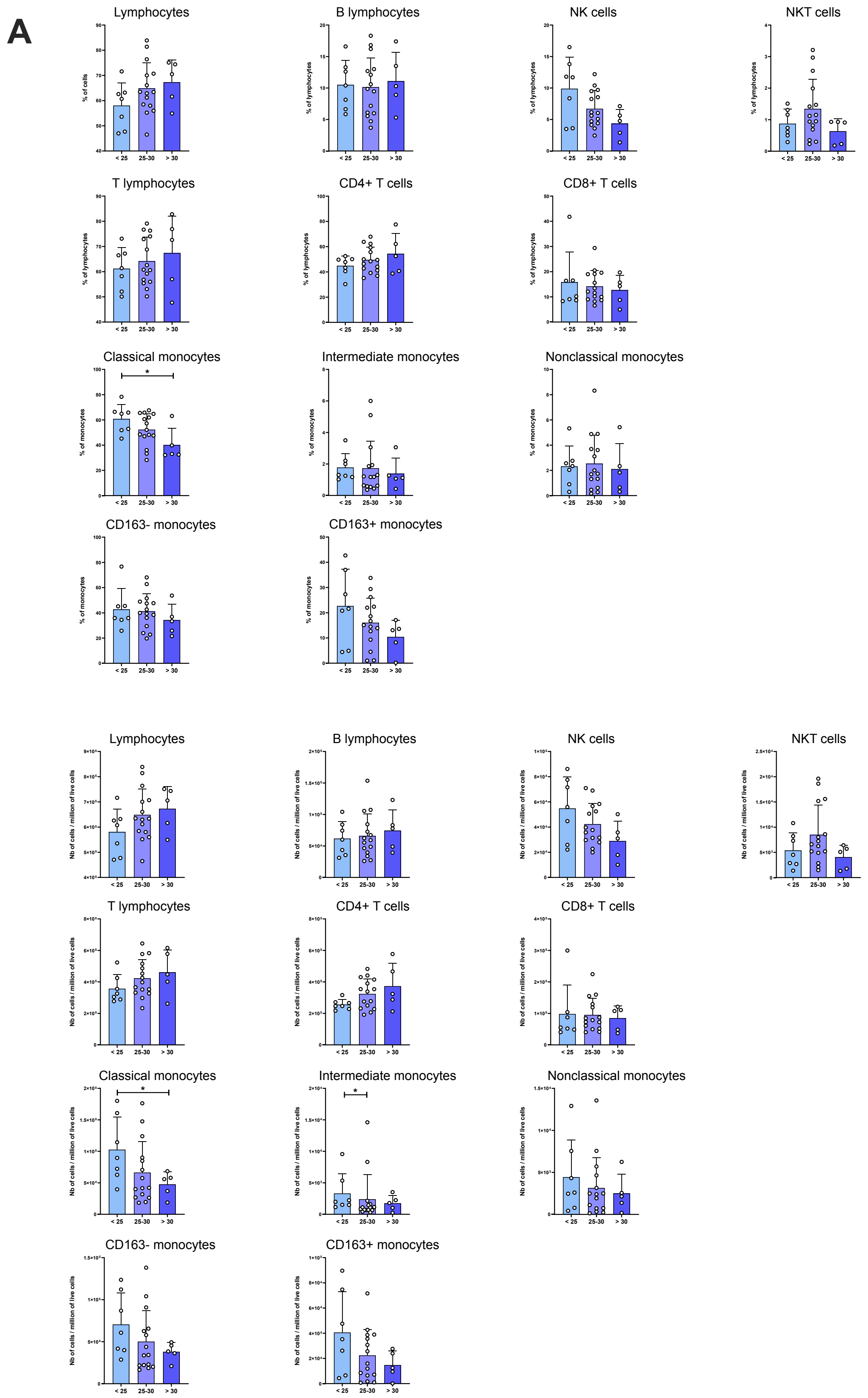
A
Lymphocytes
B lymphocytes
NK cells
NKT cells
T lymphocytes
CD4+ T cells
CD8+ T cells
Classical monocytes
Intermediate monocytes
Nonclassical monocytes
CD163- monocytes
CD163+ monocytes
% of cells
% of lymphocytes
% of monocytes
Nb of cells / million of live cells
< 25
25-30
> 30
*

B

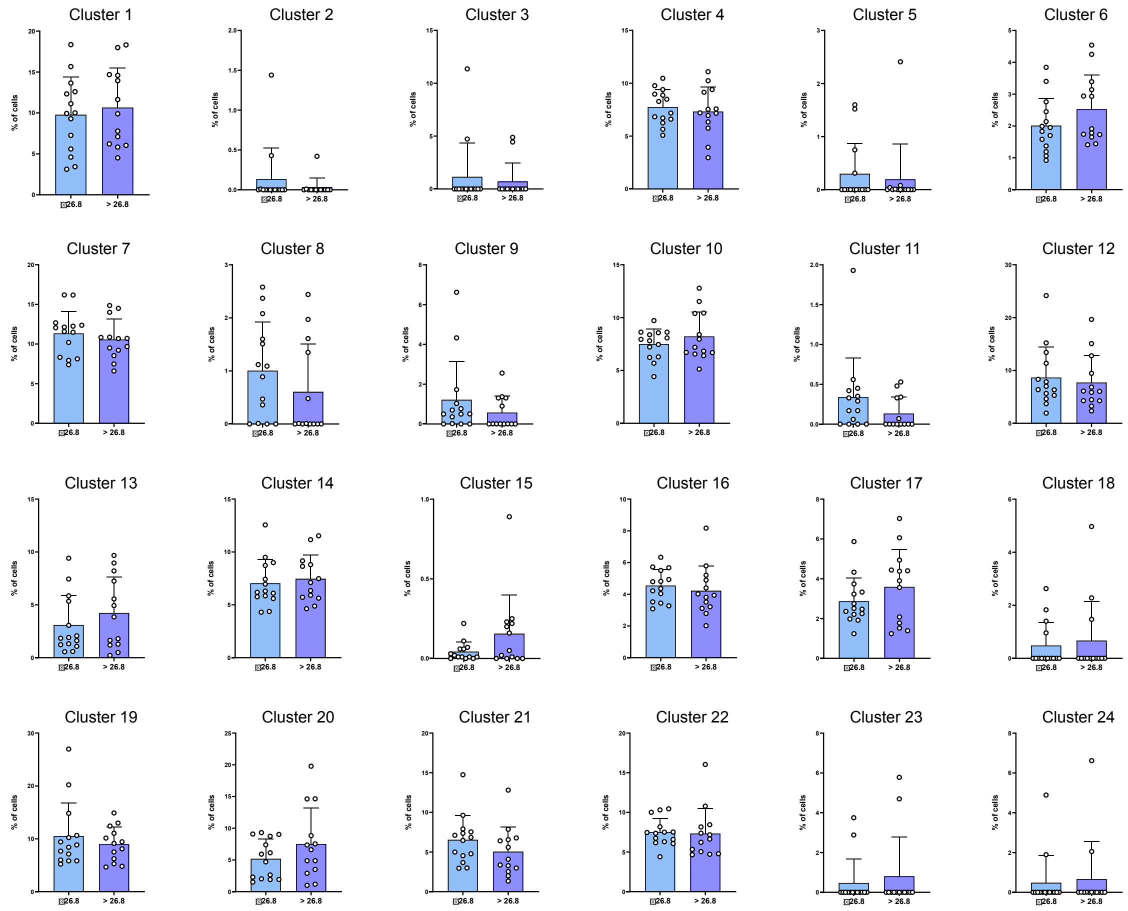

S5 Fig. (A) Percentage and absolute number (mean ± standard deviation) of lymphocytes, B lymphocytes, NK cells, NK-T cells, T lymphocytes, CD4 and CD8 T cell subsets; classical, intermediate and non-classical, CD163 and CD163 monocytes. (B) Bar plots representing the percentage of different clusters identified by Excyted pipeline in COVID-19 patients according to the BMI categories (above and below the median BMI of 26.8). Exact p value is reported if significant.
