# Supplementary material for: Differential association between inflammatory cytokines and multiorgan dysfunction in COVID-19 patients with obesity
Source: PLoS One. 2021 May 26;16(5):e0252026. doi: 10.1371/journal.pone.0252026 (PMC8153504; doi:10.1371/journal.pone.0252026)
Supplement: S7 Fig — Wilcoxon-Mann-Whitney tests used for pairwise comparisons followed by the Benjamini Hochberg test for multiple testing correction. (PDF) [file pone.0252026.s012.pdf]

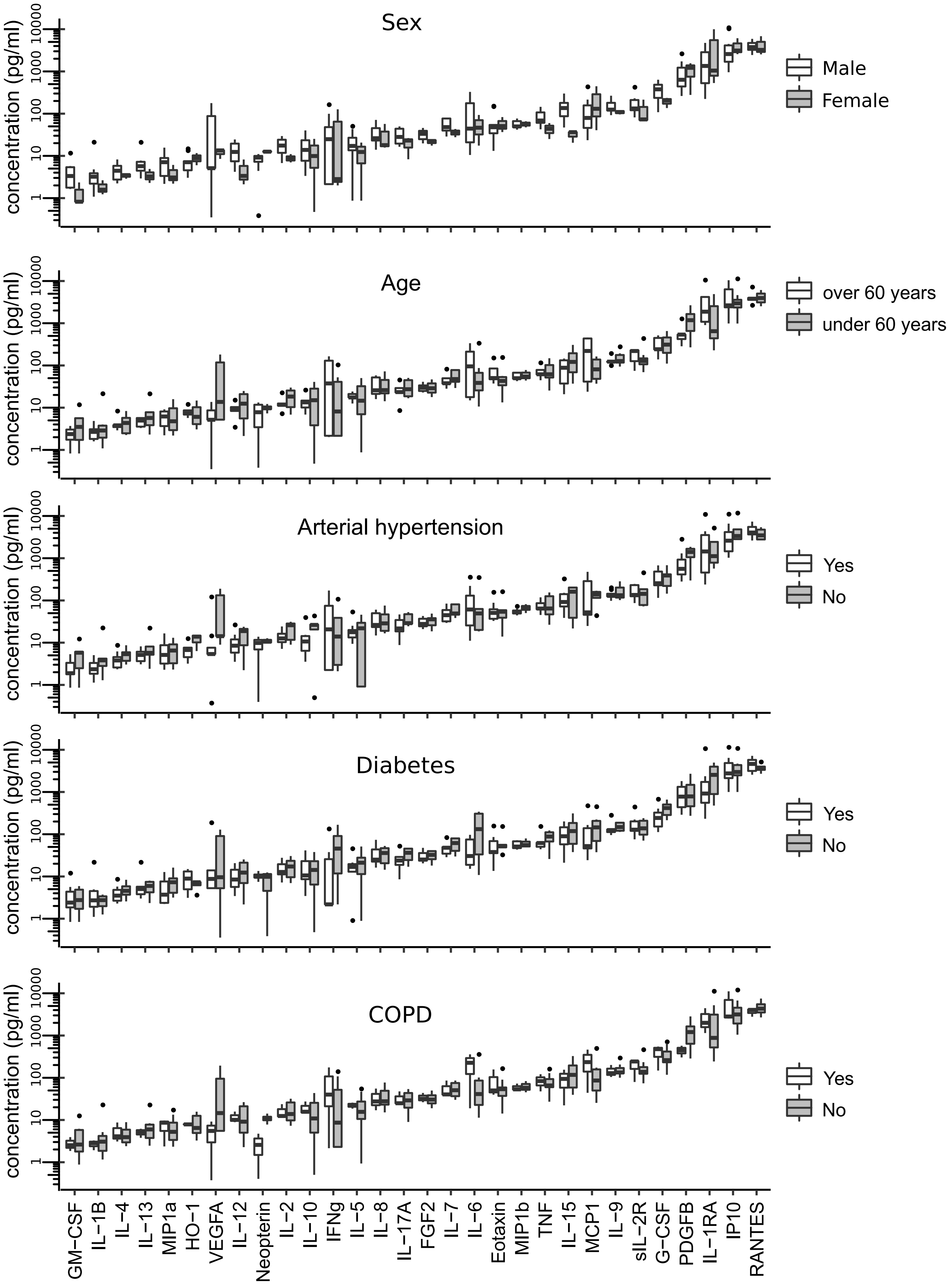

S7 Fig. Box plots illustrating the concentration of cytokines according to the sex, age, arterial hypertension, diabetes, COPD among BMI > 30 COVID-19 patients. Wilcoxon-Mann-Whitney tests used for pairwise comparisons followed by the Benjamini Hochberg test for multiple testing correction.
